# Vitamin D Status and Cause-Specific Mortality: A General Population Study

**DOI:** 10.1371/journal.pone.0052423

**Published:** 2012-12-20

**Authors:** Tea Skaaby, Lise Lotte Nystrup Husemoen, Charlotta Pisinger, Torben Jørgensen, Betina Heinsbæk Thuesen, Mogens Fenger, Allan Linneberg

**Affiliations:** 1 Research Centre for Prevention and Health, Glostrup Hospital, Glostrup, Denmark; 2 Faculty of Health Science, University of Copenhagen, Copenhagen, Denmark; 3 Faculty of Medicine, Alborg University, Alborg, Denmark; 4 Department of Clinical Biochemistry, Hvidovre Hospital, Hvidovre, Denmark; University of Tennessee, United States of America

## Abstract

**Background:**

Vitamin D deficiency is associated with an increased risk of all-cause mortality in observational studies. The specific causes of death underlying this association lack clarity. We investigated the association between vitamin D status and cause-specific mortality.

**Methods:**

We included a total of 9,146 individuals from the two population-based studies, Monica10 and Inter99, conducted in 1993–94 and 1999–2001, respectively. Vitamin D status was assessed as serum 25-hydroxyvitamin D. Information on causes of death was obtained from The Danish Register of Causes of Death until 31 December 2009. There were a total of 832 deaths (median follow-up 10.3 years).

**Results:**

Multivariable Cox regression analyses with age as underlying time axis and vitamin D quartiles showed significant associations between vitamin D status and death caused by diseases of the respiratory system, the digestive system, and endocrine, nutritional and metabolic diseases with hazard ratios (HRs) 0.26 (p_trend_ = 0.0042), 0.28 (p_trend_ = 0.0040), and 0.21 (p_trend_ = 0.035), respectively, for the fourth vitamin D quartile compared to the first. We found non-significantly lower HRs for death caused by mental and behavioural diseases and diseases of the nervous system, but no association between vitamin D status and death caused by neoplasms or diseases of the circulatory system.

**Conclusion:**

The associations of vitamin D status and cause-specific mortality suggest that we also look elsewhere (than to cardiovascular disease and cancer) to explain the inverse association between vitamin D status and mortality.

## Introduction

Vitamin D is an essential regulator of calcium homeostasis and bone mineralization; deficiency causing rickets, osteomalacia and osteoporosis. A growing body of evidence, however, indicates a broader role of vitamin D in various biological processes. The hormonally active form of vitamin D, 1,25-dihydroxyvitamin-D, has a diverse array of biological functions ranging from antiproliferative and antiangiogenic effects to modulation of the immune system [1].

Vitamin D is produced in sun exposed skin and retrieved from the diet and dietary supplements. It is metabolized in the liver and kidneys to its active form [1]. Even if dietary intake is low, the skin has the capability to produce adequate amounts of vitamin D given enough sunlight but due to our indoor lifestyle, and use of sunscreens, it often does not suffice; vitamin D deficiency is common worldwide [1]–[3].

An inverse association between vitamin D status and all-cause mortality has been established in prospective studies [4]; [5]. A meta-analysis of vitamin D supplementation reported a decrease in mortality, whereas a recent one found a reduced mortality with vitamin D and calcium supplementation in the elderly but no effect of vitamin D supplementation alone [6]; [7]. The specific causes of death underlying the association with mortality lack clarity. Vitamin D deficiency is in observational studies associated with an increased risk of cancers such as colorectal cancer [8], lung cancer [9], and breast cancer [10]; cardiovascular disease (CVD) [11]; endocrine and metabolic diseases such as diabetes mellitus [12], obesity [13], and metabolic syndrome [14]; respiratory disease such as chronic obstructive pulmonary disease (COPD) [15]; [16], and respiratory infections [17]; diseases of the digestive system such as liver disease [18]; [19], inflammatory bowel disease [20], and celiac disease [20]; and mental disorders such as dementia [21]; [22], and depression [23]. To the best of our knowledge, no studies have investigated the association of vitamin D status and different specific causes of death in the same study population.

We investigated the prospective association between vitamin D status as assessed by serum 25-hydroxyvitamin D (25-OH-D) and cause-specific mortality according to The International Classification of disease in two cohorts from a general Danish population.

## Methods

### Ethics Statement

Participants gave their written informed consent, and the studies were approved by the local ethics committee of Copenhagen County and the Danish Data Protection Agency. The recommendations of the Declaration of Helsinki were followed.

### Study Populations

We used the two population based studies, Monica10 and Inter99. The Monica10 study was a 10 year follow-up of the MonicaI study which was conducted in 1982–1984 and included examinations of 3,785 individuals. The MonicaI population was recruited from the Danish Central Personal Register as an age- and sex-stratified random sample of the population. Of the 3785 persons re-invited to the Monica10 study, 70.2% chose to participate; the study included 2,656 individuals between the ages 40–71 years and was carried out in 1993–1994 [24]. A total of 2,649 participants from the Monica10 study with measurements of vitamin D status were included in the study.

The Inter99 study carried out in 1999–2001 included 6,784 individuals aged 30–60 years drawn from an age- and sex-stratified random sample of the population in the same area as MonicaI [25]. The baseline participation rate was 52.5%. The Inter99 study was a population-based randomized controlled trial (CT00289237, ClinicalTrials.gov) investigating the effects of lifestyle intervention on CVD. Details on the study and the intervention program are described elsewhere [25]. To summarise, participants were pre-randomized into two groups, A and B, and given a lifestyle consultation with personal health advice. Participants from group A at high risk of ischemic heart disease (IHD) (60%) were furthermore offered group-based lifestyle counseling on smoking cessation, increased physical activity, and healthier dietary habits. Participation at baseline was associated with non-smoking, and fewer admissions for CVD and diabetes. A total of 6,497 participants from Inter99 with measurements of vitamin D were included in the study. Inter99 data were considered observational, and analyses were adjusted for study group.

In both studies, the health examination included a self-administered questionnaire, a physical examination, and various blood tests. The questionnaires provided information on education, intake of fish, leisure time physical activity, smoking habits, and alcohol consumption. Height and weight were measured without shoes and with light clothes. Body mass index (BMI) was calculated as weight (kg) divided by height (m) squared. Blood was drawn after an overnight fast.

### Exposure

Serum samples from the participants in the Monica10 and Inter99 studies had been stored at −20°C until the analyses of 25-OH-D in 2009 and 2010, respectively. Measurements of serum 25-OH-D in the Monica10 population were carried out by chemiluminescence immunoassay IDS-iSYS (Immunodiagnostic Systems, Boldon Tyne & Wear, UK) traceable to company standard UV/LC-MS-MS [26]. Samples were pre-treated to denature the vitamin D binding protein and neutralised in assay buffer. A specific anti-25-OH-D acridinium-labelled antibody was added. After an incubation step, magnetic particles linked to 25-OH-D were added. Following a further incubation step, a magnet captured the magnetic particles. After washing and addition of trigger reagents, the light emitted by the acridinium-label was inversely proportional to the concentration of 25-OH-D in the original sample. CV%: 16.9% (level 16.7), 8.9% (level 185.2). Specificity: 25-OH-D2 100%, 25-OH -D3 100%, co-determines 1,25-(OH)_2_-D3>100%. Detection limit was 9.0 nmol/l [27].

In Inter99, analyses of 25-OH-D were performed by high performance liquid chromatography (HPLC). Serum samples were deproteinized with acetonitrile after addition of 1-alfa-D3-vitamin as internal standard. A 90% heptan/10% dichlormethane mixture was added and thoroughly mixed. The upper organic phase was evaporated after centrifugation, and the samples were solubilized in acetonitrile. Samples were analyzed using a Phenomenex LUNA C18 3µm column and the mobile phase 90% methanol in water for separation and a Dionex HPLC equipment with a diode array detector at 267 nm for detection. The 25-OH-D2 and 25-OH-D3 were eluted at the same time, but as the absorption coefficient is the same for the two metabolites, the measured values reflect the biological active vitamin D in serum. The detection limit was 10 nmol/l [28]. The intra- and intercoefficients of variance were 7.1% and 13%, respectively.

Assessing the accuracy of the used HPLC-method, 40 serum samples were measured both by the used HPLC method and by a validated HPLC method including certified reference materials (no. 968c; NIST, Gaithersburg, Md., USA) at the National Food Institute, Technical University of Denmark (DTU) as part of the OPTIFORD-project and compared [29].

### Registry-based Diagnoses

All residents in Denmark have a unique and permanent personal civil registration number allowing linkage of data from complete national registers on an individual level. The Danish Registry of Causes of Death provided up to three diagnoses suspected to be the cause of death [30]. Denmark never used the ICD-9 but went directly from the ICD-8 to the ICD-10 before the studies took place. Consequently, the major groups of diagnoses of death were (all ICD-10): neoplasms, C00–D48; endocrine, nutritional and metabolic diseases, E00–E90; mental and behavioural disorders, F00–F99; diseases of the nervous system, G00–G99; diseases of the circulatory system, I00–I99; diseases of the respiratory system, J00–J99; and diseases of the digestive system, K00–K93. The remaining deaths (n = 96) consisted mainly of deaths caused by “Symptoms, signs and abnormal clinical and laboratory findings, not elsewhere classified” (R) and external causes of morbidity and mortality (X). Participants were followed until 31 December 2009.

### Statistical Analyses

The analyses were performed with SAS, version 9.2 (SAS Institute Inc. Cary, NC USA). All p-values were two-sided, and p-values <0.05 were considered statistically significant. Descriptive characteristics of the participants presented as percent or median according to vitamin D quartiles were compared with non-parametric statistics (table 1). Vitamin D status according to the main causes of death is presented in table 2.

**Table 1:** Baseline characteristics stratified by vitamin D quartiles (N = 9,146).

Serum 25-hydroxyvitamin D quartiles#	1^st^ quartile% or *median*	2^nd^ quartile% or *median*	3^rd^ quartile% or *median*	4^th^ quartile% or *median*	P-valueChisq/*Kruskal wallis*
Characteristics					
Age (years)	*49.8*	*49.8*	*49.8*	*49.8*	*0.79*
Male gender	49.8	51.1	49.5	47.6	0.12
Alcohol (drinks/week)					
0	13.7	10.9	9.8	9.4	**<0.0001**
< = 7	43.0	44.8	44.6	43.6	
< = 14	18.6	21.6	23.1	23.5	
>14	24.8	22.7	22.5	23.5	
No education	22.1	19.9	18.2	16.3	**<0.0001**
Body mass index (kg/m^2^)	*26.0*	*25.7*	*25.6*	*25.0*	***<0.0001***
Season (blood sample)					
Mar-May	34.6	26.8	23.7	16.6	**<0.0001**
Jun-Aug	17.4	18.3	20.5	34.4	
Sep-Nov	18.8	29.6	36.8	39.5	
Dec-Feb	29.3	25.4	19.0	9.5	
Physical activity					
Sedentary	27.0	21.5	19.3	18.2	
Light	59.6	60.7	61.5	59.3	
Moderate/vigorous	13.5	17.9	19.2	22.5	**<0.0001**
Eats fish <twice a week	66.2	64.7	64.4	64.2	0.47
Smoking habits					
Never	30.3	32.6	34.2	34.0	**<0.0001**
Former	21.5	26.7	27.4	28.5	
Current, <15 g/day	13.1	12.4	11.9	11.5	
Current, > = 15–25 g/day	22.5	19.2	17.1	16.8	
Current, >25 g/day	8.9	5.6	5.9	5.2	
Occasional	3.8	3.6	3.5	4.0	

# Ranges (median, min, max) for Inter99∶1^st^ quartile (23, 10, 32), 2^nd^ quartile (40, 33, 47), 3^rd^ quartile (55, 48, 64), 4^th^ quartile (80, 65, 255).

Ranges (median, min, max) for Monica10∶1^st^ quartile (35, 13, 45), 2^nd^ quartile (53, 45, 61), 3^rd^ quartile (70, 61, 81), 4^th^ quartile (96, 81, 204).

Data from the Monica10 and Inter99 studies were merged. Multivariate Cox regression analysis was used to determine the association of baseline vitamin D status and cause-specific mortality. Vitamin D levels were divided into quartiles before merging the two studies to account for the different methods of measuring vitamin D and different storage time. The first (lowest) quartile was used as reference. Estimates are presented as hazard ratios, HRs (95% confidence intervals, CIs). For each outcome, only participants with complete information on all considered variables were included.

We used age as underlying time axis and delayed entry. Participants were followed and contributed with risk time in the analyses until date of death, date of emigration or 31 December 2009, whichever came first. We checked the proportional hazards assumption; time-dependent covariates; and Schoenfeld residuals.

In model 1 (table 3), we adjusted for study group (no intervention [participants from Monica10], lifestyle counseling [group B from Inter99], lifestyle and group counseling [group A from Inter99]). Model 2 was further adjusted for gender; education/vocational training (no education, education including students); season of blood sample (March-May, June-August, September-November, or December-February); intake of fish (<twice a week, ≥ twice a week); physical activity during leisure time (sedentary, light, or moderate/vigorous); smoking habits (never smokers, ex-smokers, occasional smokers, current smokers <15 g/day; 15-<25 g/day, or ≥25 g of tobacco/day; 1 cigarette = 1 g, 1 cheroot = 2 g, 1 cigar = 3 g, pipe = stated in g); BMI [31] (underweight <18.5 kg/m^2^, normal weight ≥18.5–25 kg/m^2^, overweight ≥25–30 kg/m^2^, and obese ≥30 kg/m^2^); and alcohol consumption (0, >0–7, >7–14, or >14 drinks per week). To allow the effect of vitamin D on the outcomes to differ between the study groups, we checked the interaction between study group and vitamin D status (non-significant in all models). Likewise, the interaction between vitamin D status and BMI was non-significant in all models.

In supplemental analyses, we excluded participants with a diagnosis of respiratory disease (ICD-8∶240–279, ICD-10: J00–J99), digestive disease (ICD-8∶52–57, ICD-10: K00–K93), or endocrine, nutritional or metabolic disease (ICD-8∶240–279, ICD-10: E00–E90) before baseline to reduce the risk of reverse causation.

## Results

The baseline characteristics of the study populations stratified by vitamin D quartiles are shown in table 1. In crude baseline analyses, vitamin D status was associated with alcohol consumption, education, BMI, season of blood test, physical activity, and smoking habits. The median vitamin D levels at baseline were 61.0 nmol/l (interquartile range, IQR, 44.7–80.9 nmol/l) and 48.0 nmol/l (IQR 32.0–65.0 nmol/l) in the Monica10 and Inter99 studies, respectively.

Most deaths were caused by neoplasms and diseases of the circulatory system accounting for approximately 40 percent, and one fourth of deaths, respectively (table 2). Vitamin D status was lowest among the persons dying of endocrine, nutritional and metabolic diseases, mental and behavioural disorders, and diseases of the digestive system, and highest among the persons dying of diseases of the nervous system or the circulatory system (table 2). The median follow-up time was approximately 10 years.

**Table 2:** Main causes of death during follow-up in the Monica10 and Inter99 studies and vitamin D status.

Cause of death (number of events)	% (number ofevents)	25-OH-D, nmol/lMedian (p25, p75)
**Neoplasms C00–D48**	40.6 (338)	51.5 (39.0, 71.9)
Malignant neoplasms, digestive organs C15–C26	(96)	53.2 (38.3, 73.7)
Malignant neoplasm of bronchus and lung C34	(89)	50.0 (39.9, 69.0)
Malignant neoplasm of breast C50	(31)	48.0 (33.7, 74.2)
Other diagnoses included: malignant neoplasms, lip, oral cavity and pharynx C00–C14 (12); malignantneoplasms, female genital organs C51–C58 (15); malignant neoplasms, male genital organs C60–C63(13); malignant neoplasms, urinary organs C64–C68 (17); malignant neoplasms, eye, brain and centralnervous system C69–C72 (13); malignant neoplasms of lymphoid, haematopoietic and related tissueC81–C96 (19)		
**Endocrine, nutritional and metabolic diseases E00–E90**	2.0 (17)	42.8 (32.4, 52.2)
Diabetes mellitus E10–E14	(13)	42.8 (32.7, 52.2)
**Mental and behavioural disorders F00–F99**	3.6 (30)	46.5 (27.0, 76.9)
Dementia F00–F03	(12)	69.3 (43.9, 87.3)
Mental and behavioural disorders due to use of alcohol F10	(16)	33.5 (22.0, 65.4)
**Diseases of the nervous system G00–G99**	2.6 (22)	54.7 (40.0, 77.4)
Diagnoses included: Alzheimer’s disease G30 (8)		
**Diseases of the circulatory system I00–I99**	26.6 (221)	58.0 (41.0, 76.3)
Ischemic heart diseases I20–I25	(90)	60.3 (42.3, 78.5)
Cerebrovascular diseases I60–I69	(62)	53.2 (38.4, 76.3)
Other diagnoses included: hypertensive diseases I10–I15 (8); heart failure I50 (9); diseases of arteries, arterioles and capillaries I70–I79 (13)		
**Diseases of the respiratory system J00–J99**	7.9 (66)	50.6 (31.3, 62.6)
Respiratory infections J00–J22	(11)	47.8 (41.3, 62.6)
Chronic obstructive pulmonary disease J44	(42)	51.0 (31.0, 60.5)
**Diseases of the digestive system K00–K93**	5.1 (42)	44.1 (31.5, 58.2)
Diseases of liver K70–K77	(23)	43.2 (27.0, 57.0)
Other diagnoses included: diseases of esophagus, stomach and duodenum K20–K31 (7)		

Abbreviations: 25-OH-D; 25-hydroxyvitamin D.

We found significant inverse associations between vitamin D status and death caused by diseases of the respiratory system, the digestive system, and endocrine, nutritional and metabolic diseases (table 3). The estimates were stable through multiple adjustments. The hazard ratio for the fourth vitamin D quartile with the first quartile as reference was approximately 0.25 for all three groups.

**Table 3:** Hazard ratios and 95% confidence intervals for the prospective associations between serum 25-OH vitamin D status and cause-specific mortality (individuals included = 8,329, person years at risk = 85,719).

Death caused by	Events	Model 1$HR (95% CI)	Model 2&HR (95% CI)
Neoplasms	301		
1^st^ vitamin D quartile		1 (reference)	1 (reference)
2^nd^ vitamin D quartile		1.1 (0.74, 1.4)	1.1 (0.82, 1.5)
3^rd^ vitamin D quartile		0.90 (0.65, 1.2)	1.1 (0.78, 1.5)
4^th^ vitamin D quartile		0.68 (0.48, 0.95)	0.81 (0.57, 1.2)
		P_trend_ = **0.020**	P_trend_ = 0.29
Endocrine, nutritional and metabolic diseases	15		
1^st^ vitamin D quartile		1 (reference)	1 (reference)
2^nd^ vitamin D quartile		0.35 (0.09, 1.3)	0.37 (0.09, 1.4)
3^rd^ vitamin D quartile		0.23 (0.05, 1.09)	0.23 (0.05, 1.2)
4^th^ vitamin D quartile		0.24 (0.05, 1.1)	0.21 (0.04, 1.1)
		P_trend_ = **0.030**	P_trend_ = **0.035**
Mental and behavioural disorders	21		
1^st^ vitamin D quartile		1 (reference)	1 (reference)
2^nd^ vitamin D quartile		0.20 (0.04, 0.93)	0.20 (0.04, 0.96)
3^rd^ vitamin D quartile		0.38 (0.12, 1.2)	0.37 (0.11, 1.3)
4^th^ vitamin D quartile		0.57 (0.20, 1.6)	0.44 (0.14, 1.4)
		P_trend_ = 0.35	P_trend_ = 0.20
Diseases of the nervous system	20		
1^st^ vitamin D quartile		1 (reference)	1 (reference)
2^nd^ vitamin D quartile		0.60 (0.17, 2.1)	0.67 (0.18, 2.5)
3^rd^ vitamin D quartile		0.68 (0.21, 2.2)	0.76 (0.22, 2.6)
4^th^ vitamin D quartile		0.74 (0.23, 2.5)	0.75 (0.21, 2.7)
		P_trend_ = 0.68	P_trend_ = 0.71
Diseases of the circulatory system	178		
1^st^ vitamin D quartile		1 (reference)	1 (reference)
2^nd^ vitamin D quartile		0.63 (0.41, 0.99)	0.69 (0.44, 1.1)
3^rd^ vitamin D quartile		0.91 (0.61, 1.3)	1.1 (0.74, 1.7)
4^th^ vitamin D quartile		0.85 (0.56, 1.3)	1.1 (0.70, 1.7)
		P_trend_ = 0.80	P_trend_ = 0.35
Diseases of the respiratory system	47		
1^st^ vitamin D quartile		1 (reference)	1 (reference)
2^nd^ vitamin D quartile		0.93 (0.47, 1.8)	0.95 (0.47, 1.9)
3^rd^ vitamin D quartile		0.47 (0.21, 1.1)	0.47 (0.20, 1.1)
4^th^ vitamin D quartile		0.29 (0.11, 0.78)	0.26 (0.09, 0.75)
		P_trend_ = **0.0035**	P_trend_ = **0.0042**
Diseases of the digestive system	34		
1^st^ vitamin D quartile		1 (reference)	1 (reference)
2^nd^ vitamin D quartile		0.66 (0.29, 1.5)	0.64 (0.28, 1.5)
3^rd^ vitamin D quartile		0.26 (0.08, 0.78)	0.24 (0.08, 0.74)
4^th^ vitamin D quartile		0.39 (0.15, 1.0)	0.28 (0.10, 0.78)
		P_trend_ = **0.014**	P_trend_ = **0.0040**

$ Participants with missing values in one of the used variables were excluded. Adjusted for study group.

& Further adjusted for gender, education, season of blood sample, intake of fish, physical activity, smoking habits, body mass index, and alcohol consumption.

Abbreviations: CI, confidence interval; HR, hazard ratio; 25-OH vitamin D; 25-hydroxyvitamin D.

In model 1, we found a significant inverse linear trend between vitamin D quartiles and death caused by neoplasms. The association, however, was no longer significant in the fully adjusted model when further adjusting for intake of fish, physical activity, smoking habits, body mass index, and alcohol consumption.

We found non-significantly lower HRs for mental and behavioural diseases and diseases of the nervous system. However, we found no association between vitamin D status and death caused by diseases of the circulatory system.

In additional analyses (data not shown) of the three major causes of death showing significant associations with vitamin D status, we excluded participants with a baseline diagnosis of the disease. Thus, we excluded persons with baseline respiratory disease (n = 929) for the outcome “death caused by diseases of the respiratory system”, persons with baseline digestive disease (n = 1,367) for the outcome “death caused by diseases of the digestive system, and persons with baseline endocrine, nutritional and metabolic diseases (n = 408) for the outcome “death caused by endocrine, nutritional and metabolic diseases”. The estimates remained stable for all three outcomes but for death caused by endocrine, nutritional and metabolic diseases, the association was no longer statistically significant.

## Discussion

To the best of our knowledge, no studies have investigated the association of vitamin D status and different specific causes of death in the same study population. We found a significant inverse association between vitamin D status and death caused by diseases of the respiratory and the digestive system and by endocrine, nutritional and metabolic diseases, but no association with death caused by neoplasms or diseases of the circulatory system.

Our results regarding an inverse association between vitamin D status and death caused by respiratory disease are in line with previous studies. Romme et al found that vitamin D deficiency was present in the majority of patients with COPD entering pulmonary rehabilitation [15], and Black et al found a dose-response relationship between the serum concentration of 25-hydroxyvitamin D and FEV_1_ [16]. Likewise, a study by Ginde et al found an inverse association between 25-OH-D levels and recent upper respiratory tract infections [17]. Vitamin D could affect respiratory disease in a number of ways: it inhibits the formation of matrix metalloproteinases as well as fibroblast proliferation, and influences collagen synthesis thereby possibly influencing tissue remodeling [32]. In addition, it is suggested that vitamin D plays an important role in innate immunity, particularly through the antimicrobial peptide cathelicidin [33]. Since low BMI is an independent risk factor for mortality in subjects with COPD [34], it may be speculated whether vitamin D status is just a reflection of the level of malnutrition. The association, however, remained significant after adjusting for BMI and also after excluding the participants with a diagnosis of respiratory disease before the study, thereby providing some support for a possible causal effect of vitamin D on respiratory disease.

The observed inverse association between vitamin D status and death caused by digestive disease –liver disease accounting for over half– is consistent with previous findings of a high prevalence of vitamin D deficiency in patients with liver disease [18], and Putz-Bankuti et al found that low 25-OH-D levels predicted hepatic decompensation and mortality in patients with chronic liver failure [35]. Vitamin D deficiency might contribute to liver damage by increased inflammation and fibrosis and by reduced antiviral response [20]; [36]–[38]. Barchetta et al proposed a causal role of vitamin D in the development of non-alcoholic fatty liver disease by exerting a dose-dependent effect on fat accumulation into the hepatocytes [39].

However, the low vitamin D levels associated with liver disease could be a consequence of the disease rather than a cause. Patients with digestive disease are particularly prone to vitamin D deficiency due to fat malabsorption; bile salt deficiency; loss of absorptive surface; increased intestinal permeability; loss of liver function causing impaired hepatic hydroxylation of vitamin D, reduced hepatic production of vitamin D binding protein (DBP); or an impaired cutaneous production due to reduced sun exposure or jaundice [18]; [20]. The total levels of 25-OH-D are highly correlated with levels of DBP and albumin, and adjusting for liver function would have been an advantage. We attempted to reduce the bias of reverse causation by excluding the persons with a diagnosis of digestive disease (including liver disease) before the study. The association remained significant, strengthening our belief in a causal relationship between vitamin D and development of liver disease.

The observed lack of association between vitamin D status and circulatory disease mortality supports the results from a study by Melamed et al [40]. It is, however, inconsistent with several other observational studies [4]; [41]; [42], including a recent study by Thomas et al who found an optimal vitamin D level to lower all-cause and cardiovascular disease mortality in patients with the metabolic syndrome [43]. There may be several reasons for the different results. Most of the mentioned studies examined older people, or subgroups, where vitamin D could be more important regarding CVD risk. Worth mentioning, two studies of vitamin D supplementation found no effect on CVD [44]; [45].

Our results regarding an association between vitamin D status and death caused by endocrine, nutritional and metabolic diseases – diabetes mellitus accounting for three fourths – conform with results from previous observational studies [12]; [46]. The possible mechanisms are uncertain but vitamin D may preserve glucose tolerance through effects on insulin secretion and sensitivity [47].

The observed lack of association between vitamin D status and death caused by neoplasms is consistent with a study by Freedman et al reporting no overall relationship between 25-OH-D and cancer mortality risk in the general population [48]. However, despite a proposed role for vitamin D in some of the common pathways of cancer –i.e. through induction of apoptosis and prevention of angiogenesis in malignant cells, thereby reducing the potential for the malignant cell to survive [1]– different types of cancer may not share the same possible vitamin D dependent pathway. A meta-analysis by Ma et al thus reported an inverse association between blood 25-OH-D levels and the risk of colorectal cancer [8], whereas a meta-analysis on 25-OH-D levels and breast cancer by Yin et al found no significant association in the cohort studies measuring vitamin D status at baseline before cancer diagnosis [49]. As yet, results regarding a potential role for vitamin D in the prevention of cancer are conflicting [50].

Although non-significant, the observed inverse association between vitamin D status and mental and behavioural disorders, is consistent with a previous study by Annweiler et al reporting an inverse association between vitamin D intake and risk of Alzheimer’s disease [21] and a study by Hogberg et al showing amelioration of depressive symptoms after vitamin D supplementation in 54 depressed adolescents [23]. On the one hand, low cognitive function may lead to low dietary intakes of vitamin D and a lack of sunlight exposure leading to lower vitamin D levels. On the other hand, since vitamin D is a steroid hormone exerting neurosteroid actions in the central nervous system, it may partially explain neuronal degeneration and dementia [51]. Interestingly, recent studies found yet a significant positive association of vitamin D with cognition after adjusting for nutritional and physical status [51].

As mentioned above, some of the diseases are likely to cause rather than be caused by the low vitamin D status. Possible examples include liver disease and mental disease caused by alcohol abuse which may also have reduced liver function. Likewise, vitamin D could simply be a marker of a healthy lifestyle as shown in table 1. Thus, the associations may at least be explained partly by residual confounding.

The strengths of our study include the longitudinal population-based design and the large random sample of the Danish population highly prevalent of low vitamin D status [28]; a long-term follow-up and the use of standardised registry-based diagnoses with almost no individuals lost to follow-up; and the available information on potential confounders.

The limitations of the study include the loss of information when merging studies; the different methods of vitamin D measurements in the merged studies; the non-specific nature of the main causes of death; the interventional design of the Inter99 study; the lack of objective markers of liver and kidney function; and the single vitamin D measurement. A single 25-OH-D measurement may lose predictive power over time. Thus, Grant found a diminishing utility over time of a single measurement of 25-OH-D for determining the effect of vitamin D in reducing the risk of cancer [52]. Somewhat supportive of the use of a single 25-OH-D measurement to predict future health outcomes, Jorde et al found vitamin D status to be as stable a predictor as blood pressure and lipids [53], but the findings are likely an underestimate of the true associations. The mortality is generally low in this general population sample with median age just below 50 years giving a low number of events in some of the major causes of death, especially among the non-cardiovascular and non-neoplasm groups. Thus, the power for statistical analysis in most of these categories is low.

In conclusion, we found significant inverse associations between vitamin D status and death caused by diseases of the endocrine, the respiratory, and the digestive system but no associations with death caused by neoplasms or diseases of the circulatory system. Due to the explorative nature of the study and the low number of events in some of the disease categories, the results need to be confirmed in other studies. The results, however, suggest that we also look elsewhere (than to cardiovascular disease and cancer) to explain the inverse association between vitamin D status and mortality.
